# Alcohol consumption, blood DNA methylation and breast cancer: a Mendelian randomisation study

**DOI:** 10.1007/s10654-022-00886-1

**Published:** 2022-06-16

**Authors:** Xuan Zhou, Lili Yu, Lijuan Wang, Jiarui Xiao, Jing Sun, Yajing Zhou, Xiaolin Xu, Wanghong Xu, Athina Spiliopoulou, Maria Timofeeva, Xiaomeng Zhang, Yazhou He, Haomin Yang, Harry Campbell, Ben Zhang, Yimin Zhu, Evropi Theodoratou, Xue Li

**Affiliations:** 1 Department of Big Data in Health Science School of Public Health, and Centre of Clinical Big Data and Analytics of The Second Affiliated Hospital, Zhejiang University School of Medicine, Hangzhou, Zhejiang China; 2 The Key Laboratory of Intelligent Preventive Medicine of Zhejiang Province, Hangzhou, China; 3 School of Public Health, Fudan University, Shanghai, China; 4 Centre for Population Health Sciences, Usher Institute, University of Edinburgh, Edinburgh, UK; 5 Danish Institute for Advanced Study (DIAS), Epidemiology, Biostatistics and Biodemography Research Unit, Institute of Public Health, University of Southern Denmark, Odense, Denmark; 6 Centre for Global Health, Usher Institute, University of Edinburgh, Edinburgh, UK; 7 West China School of Public Health, Sichuan University, Chengdu, Sichuan China; 8 Department of Epidemiology and Health Statistics, School of Public Health, Fujian Medical University, Fuzhou, Fujian China; 9 Department of Medical Epidemiology and Biostatistics, Karolinska Institutet, Stockholm, Sweden

**Keywords:** Alcohol, DNA methylation, Breast cancer, Mendelian randomisation

## Abstract

Alcohol intake is thought to be a risk factor for breast cancer, but the causal relationship and carcinogenic mechanisms are not clear. We performed an up-to-date meta-analysis of prospective studies to assess observational association, and then conducted MR analysis to make causal inference based on the genetic predisposition to alcohol consumption (“drinks per week”) and pathological drinking behaviours (“alcohol use disorder” and “problematic alcohol use”), as well as genetically predicted DNA methylation at by alcohol-related CpG sites in blood. We found an observational dose–response association between alcohol intake and breast cancer incidence with an additional risk of 4% for per 10 g/day increase in alcohol consumption. Genetic predisposition to alcohol consumption (“drinks per week”) was not causally associated with breast cancer incidence at the OR of 1.01 (95% CI 0.84, 1.23), but problematic alcohol use (PAU) was linked to a higher breast cancer risk at the OR of 1.76 (95% CI 1.04, 2.99) when conditioning on alcohol consumption. Epigenetic MR analysis identified four CpG sites, cg03260624 near *CDC7* gene, cg10816169 near *ZNF318* gene, cg03345232 near *RIN3* gene, and cg26312998 near *RP11-867G23.13* gene, where genetically predicted epigenetic modifications were associated with an increased breast cancer incidence risk. Our findings re-affirmed that alcohol consumption is of high risk for breast cancer incidence even at a very low dose, and the pathogenic effect of alcohol on breast cancer could be due to pathological drinking behaviour and epigenetic modification at several CpG sites, which could be potential intervention targets for breast cancer prevention.

**Supplementary Information:**

The online version contains supplementary material available at 10.1007/s10654-022-00886-1.

## Introduction

According to the latest global cancer statistics, the incidence rate of breast cancer has surpassed lung cancer ranking as first, with around 2.3 million new cases in 2020 [1]. Meanwhile, breast cancer is the fifth most common cancer death, with 685,000 deaths every year. It is estimated that in women, one in four cancer cases and one in six cancer deaths are due to breast cancer [1].

A large body of research provides evidence that alcohol consumption is associated with increased risk of breast cancer. The World Cancer Research Fund (WCRF) examined the association between alcohol drinking and breast cancer based on existing literature, and concluded that there was strong evidence for a causal role of alcohol intake in postmenopausal breast cancer and probable evidence in premenopausal breast cancer, with an additional risk of 9% per 10 g/day increase in alcohol consumption [2]. A number of studies have examined the association between alcohol consumption and breast cancer subtypes, but the findings are often incomparable or inconsistent as different subtype comparisons are evaluated in different studies [3–5]. A pooled analysis identified positive associations between alcohol consumption and the risk of breast cancer subtypes regardless of the status of two hormone receptors (i.e., estrogen receptor, ER; progesterone receptor, PR), and there was no significant difference between the hormone receptor positive or negative groups [3]. However, another pooled analysis observed slightly attenuated risk for the hormone receptor negative groups [4]. In addition, alcohol was reported to be associated with lower risk of human epidermal growth factor receptor 2 (*HER2*) enriched breast cancer relative to ER positive breast cancer [5]. Few studies have examined the association between alcohol and breast cancer by drinking behaviours (i.e., alcohol use disorder and problematic alcohol use) [6]. A comprehensive examination of drinking behaviours (i.e., drinking pattern) and breast cancer types (i.e., intrinsic-like subtypes) is needed to better understand these observational associations.

The putative causal link between alcohol consumption and breast cancer is mainly based on data from conventional observational studies, but alcohol drinking itself is associated with many other lifestyle and socioeconomic factors, which may bias the association with breast cancer. Additionally, most of the observational studies only measured the alcohol consumption once or a few times over the study period, which cannot quantify the long-term alcohol exposure. Mendelian Randomisation (MR) is a method designed to estimate the causal relationship between a modifiable environmental exposure and a medically relevant trait or disease, using genetic variants as instrumental variables (IVs). Recent meta-analyses of genome-wide association studies (GWAS) identified a number of single nucleotide polymorphisms (SNPs) associated with alcohol consumption and pathological drinking behaviours of alcohol use disorder (AUD) and problematic alcohol use (PAU)[7, 8], which represent severe alcohol dependence. These SNPs can be used as instrumental variables to proxy the genetic predisposition to different alcohol drinking behaviours in MR analyses.

Beyond causality, understanding how alcohol consumption may be modulating breast cancer risk is also important. It had been hypothesized that epigenetic modification is responsible for the pathogenic effect of alcohol on cancer. Evidence from epigenome-wide association studies (EWAS) showed that alcohol consumption can affect DNA methylation in both blood and breast tissues, and meanwhile de novo methylation was observed to be associated with tumorigenesis [9, 10]. Nevertheless, the relationship between DNA methylation and breast cancer risk is not clear. A body of studies has explored the associations between DNA methylation and breast cancer and identified multiple CpG sites that were associated with breast cancer, but these associations did not replicate in a meta-analysis of independent study populations [11]. In addition, no study has ever investigated whether alcohol related DNA methylation is causally associated with breast cancer.

In this study, we aimed to provide an up-to-date and comprehensive examination of the causal relationship between alcohol consumption and breast cancer incidence risk. Using observational data from independent population-based prospective cohorts, we firstly estimated the magnitude of the observed association between alcohol intake and the risk of breast cancer incidence. Using the two-sample MR approaches, we investigated whether there was evidence of causality for the observed association and how alcohol exerts its pathogenic effect on the incidence of breast cancer.

## Methods

### Meta-analysis of prospective studies

We carried out a comprehensive literature search for prospective studies that explored the associations between alcohol drinking and breast cancer incidence risk in MEDLINE and EMBASE databases (both from the OVID interface) from inception to March 22, 2021, using keywords of alcohol, breast cancer, and cohort. For each study included for eligibility, we set grams per day as a standard, considering one drink as 12.5 g, one ml as 0.8 g, and 1 oz as 28 g ethanol unless it had been specified in original studies. We assigned the midpoint for a range and the lower bound plus three-quarters of the length of the previous category for an open-ended upper category as the exposure level and divided them into three categories (≤ 12.5, ≤ 50 and > 50 g/day as light, moderate and heavy drinking, respectively). For each of the three categories, we performed a meta-analysis to compute a pooled RR with its 95% CI of breast cancer incidence using the inverse variance weighted random-effects model. We also conducted stratification analyses by menopausal status (i.e., premenopausal or postmenopausal), hormone receptor status of breast cancer (i.e., ER + /PR + or PR −), as well as the geographical areas where the studies were conducted (i.e., Europe, North America, and Asia). For the dose–response analysis, we assigned the transformed exposure levels as the doses and their corresponding RR or HR estimates as responses, using generalized least squares regression methods to estimate the overall and subgroup dose–response effects [12]. The detail methods of the meta-analysis can be seen in Supplementary Methods.

### Causal inference—two-sample Mendelian randomisation

#### Deriving genetic instruments for alcohol consumption

We selected genetic instruments for alcohol consumption from a GWAS conducted by the GWAS and Sequencing Consortium of Alcohol and Nicotine use (GSCAN) in 1.2 million individuals of European ancestry [7]. A total of 99 SNPs was identified as significantly associated with alcohol consumption (drinks per week) at the genome-wide level (*P* < 5 × 10^–8^) [7], among which seven SNPs were located on genes related to alcohol metabolism. To complement the analysis with pathological drinking patterns, we selected two more sub-phenotypes (AUD and PAU) as the exposure from a GWAS meta-analysis of European-ancestry individuals (Million Veteran Program, Psychiatric Genomics Consortium, and UK Biobank) [8]. A total of 30 and 42 SNPs were identified at genome-wide significance (*P* < 5 × 10^–8^) for AUD and PAU, respectively [8]. To derive an independent set of genetic instruments for each trait, we excluded SNPs in linkage disequilibrium (LD, r^2^ > 0.01), and the ones with the smallest *P* value in relation to each trait were retained. To identify genetic variants associated with AUD and PAU independently from the association with alcohol consumption, the GCTA-mtCOJO analysis was performed conditioning on alcohol consumption measured as drinks per week, which excludes the overlapping or highly correlated variants with effect estimates for drinks per week extracted from the GSCAN study [8].

#### mQTL identification

We selected blood DNA methylation markers (CpG sites) for alcohol consumption from an epigenome-wide association meta-analysis of 13 population-based cohorts of European ancestry using whole blood DNA [9]. The level of methylation was measured by the Illumina Infinium HumanMethylation450 (HM450) BeadChip array, and the associations between alcohol consumption and blood DNA methylation was adjusted for age, sex, BMI, batch effects and white cell blood counts (i.e., CD4 cells, CD8 cells, natural killer cells, B cells and monocytes) to minimize confounding effects [9]. Then, we identified the methylation quantitative traits loci (mQTLs) that regulate the methylation levels of these alcohol-related CpG sites to use as IVs from the mQTL database provided by the Accessible Resource for Integrated Epigenomic Studies (ARIES) project [13]. The ARIES project was launched using the Illumina Infinium HumanMethylation450 (HM450) BeadChip to acquire epigenetic data (CpG sites) and the Illumina Infinium Human Hap550 and 660-w quad genome-wide SNP genotyping platform to acquire genetic data (SNPs) using peripheral blood samples from the mothers of the 1018 mother–offspring pairs in the Avon Longitudinal Study of Parents and Children (ALSPAC) cohort [14–16]. The Matrix eQTL software [17] was used for preliminary association analysis of SNPs with CpG sites. SNPs with P < 1 × 10^–7^ were further analysed using exact linear regression including covariates in PLINK 1.07, and conditional analysis implemented in GCTA to determine the most representative independent loci associated with each CpG site [13]. Given that breast cancer is more common in middle-aged women, we extracted the mQTL-DNA methylation beta-coefficient (SD change in DNA methylation per minor allele) at the middle age time point of the mothers. For each of the alcohol consumption related CpG sites we used the conditional results, which were adjusted for age, estimated white blood cell counts, ancestry principal components, and bisulphite conversion batch.

#### GWAS summary-level data of female breast cancer

The Breast Cancer Association Consortium (BCAC) is an international consortium established to conduct collaborative studies in breast cancer [18]. For overall breast cancer, 133,384 cases and 113,789 controls from iCOGS, OncoArray and other published GWAS data were included for analysis; GWAS summary level data for hormone receptor negative breast cancer (i.e., *HER2*-Enriched-like and triple-negative breast cancer) including 106,278 invasive cases and 91,477 controls were also made available for subgroup analysis [19]. Additionally, triple-negative breast cancer cases in BCAC were also combined with affected carriers of *BRCA1* mutation in the Consortium of Investigators of Modifiers of BRCA1/2 (CIMBA) to increase the statistical power for the investigation of triple-negative susceptibility variants, and the combined datasets included 18,016 triple-negative breast cancer cases and 100,971 controls [19]. For each of the genetic IVs selected above for drinks per week, AUD, PAU and alcohol-related methylation, the effect estimates (change in risk of breast cancer incidence per effect allele) with their standard errors, the effect and reference alleles, and the effect allele frequency were extracted from the GWAS summary-level data of female breast cancer.

#### Two-sample Mendelian randomisation

For each genetic instrument in two-sample MR, β_GP_ refers to the estimates of the association between the genetic instrument and the exposure and β_GD_ refers to the estimates of the association between the genetic instruments and the outcome. The causal effect is estimated using the formula β_GD_/β_GP_ (Wald ratio) and combined using the inverse variance weighted (IVW) approach. To control for potential bias induced by correlated variants and weak instrument, we removed SNPs in linkage disequilibrium (r^2^ > 0.01) or F-statistic less than 10. To validate the MR assumptions, we assessed overall horizontal pleiotropy by (1) quantifying the heterogeneity of the genetic variants based on the Q statistic by using modified weights for the IVW approach, and (2) testing the intercept in the MR-Egger test [20]. To account for horizontal pleiotropy, we performed additional MR analyses using simple mode, weighted median, and weighted mode approaches as sensitivity analyses to explore the robustness of the findings in presence of any potential genetic pleiotropy [21]. As an additional control for pleiotropy, we applied the global test, outlier test, and distortion test using the MR pleiotropy residual sum and outlier (MR-PRESSO) approach [22]. Details of these MR approaches, including their different assumptions, are provided in Supplementary Methods. All analyses were carried out using the “TwoSampleMR” and “MRPRESSO” R packages [22, 23].

For the main MR analyses, we tested the causal association between genetic predisposition to alcohol drinking and breast cancer, using IVs for drinks per week, AUD, and PAU, as well as IVs for AUD and PAU conditioning on drinks per week (excluding the overlapping or highly correlated genetic IVs with drinks per week). As sensitivity analyses, we (1) selected IVs associated with alcohol metabolism only; (2) excluded IVs associated with obesity related traits (i.e., BMI, waist circumference, hip circumference, and weight), reproductive traits (i.e., age at menarche, age at menopause), smoking, education, and previous breast cancer at the threshold of 5 × 10^–8^ in European ancestry samples by querying PhenoScanner [24].

**Fig. 1:**
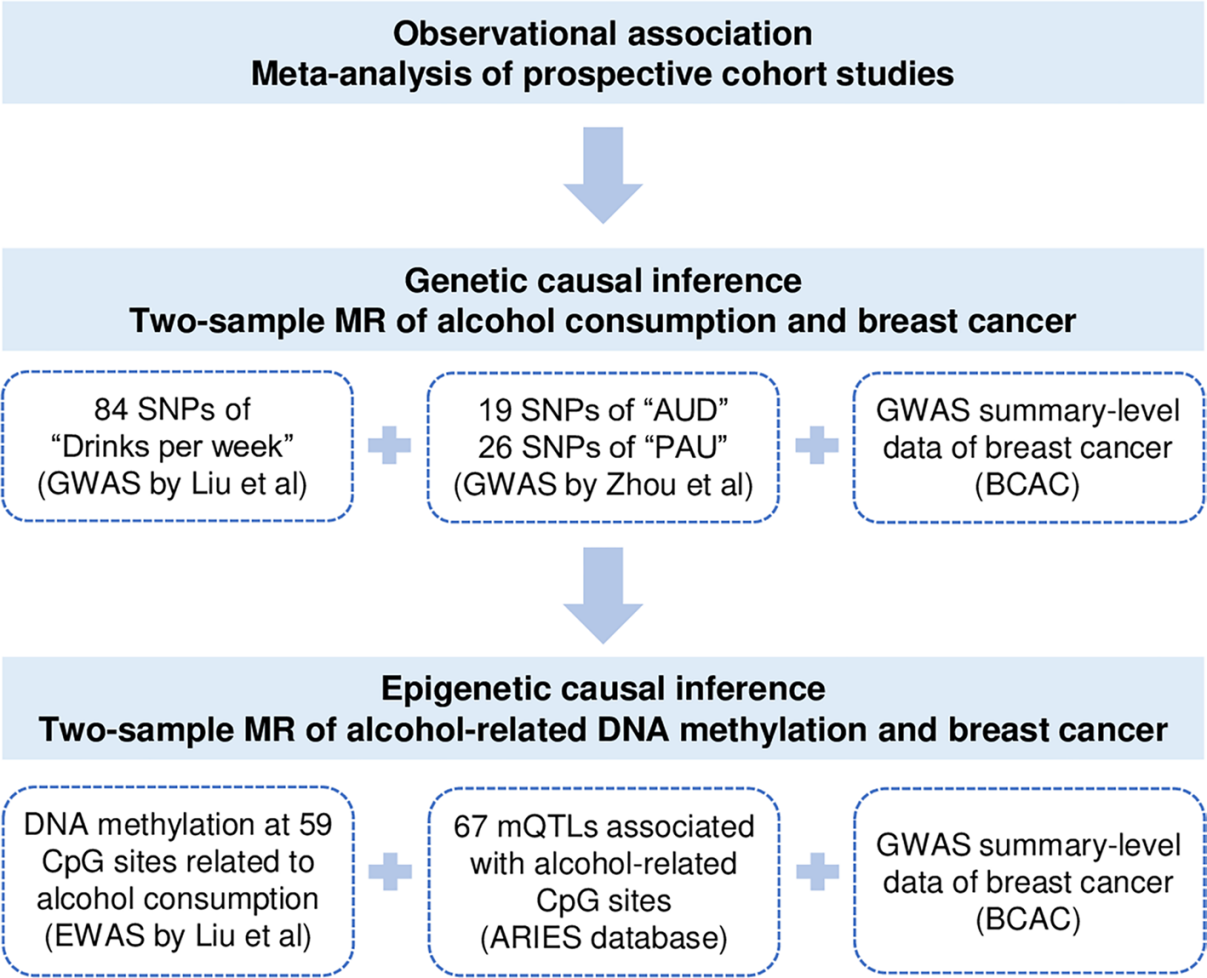
Study design

While evaluating the causal effect of genetically predicted DNA methylation in peripheral blood at alcohol related CpG sites on the risk of breast cancer incidence, each alcohol related CpG site was treated as an independent exposure, and its proxy mQTLs in the ARIES database were used as IVs. The effect allele for each mQTL was chosen so that the effect of mQTL on DNA methylation was in the same direction as the effect of alcohol intake. To control for horizontal pleiotropy, we excluded mQTLs which were associated with multiple CpG sites or associated with the aforementioned potential confounders. If there was only one mQTL for a CpG site, only the Wald ratio with its corresponding standard error was calculated. If a CpG site was associated with multiple independent mQTLs, the aforementioned IVW approach was applied. Bonferroni correction was applied to account for multiple testing. The study design and datasets used for analyses is shown in Fig. 1. All analyses were conducted using R version 4.0.3.

## Results

### Meta-analysis of prospective studies

**Fig. 2:**
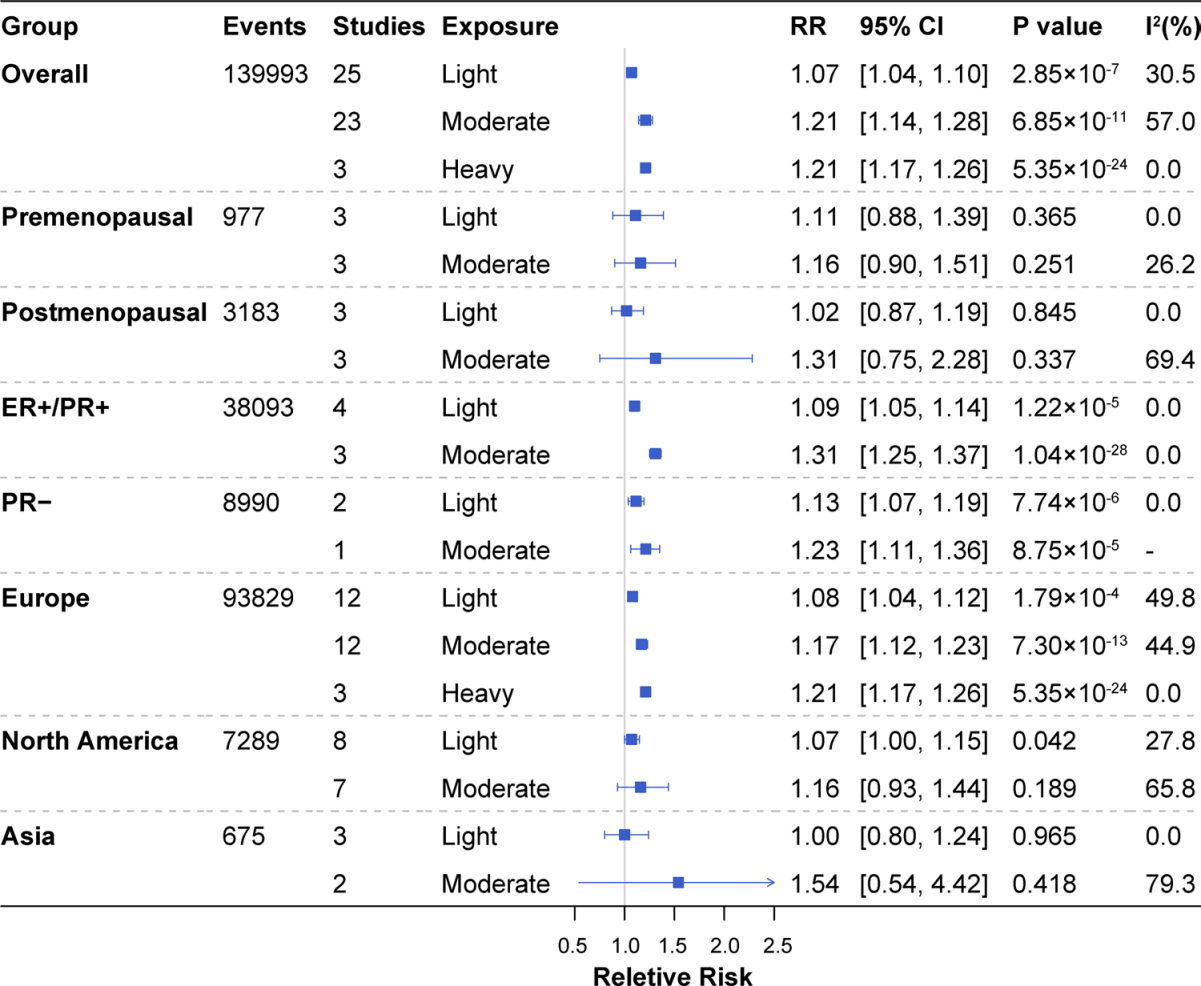
Meta-analysis of Observational RR estimates for alcohol consumption on breast cancer. RR, relative risk; 95% CI, 95% confidence interval; ER +/PR +, estrogen receptor positive and/or progesterone receptor positive; PR-, progesterone receptor negative

**Fig. 3:**
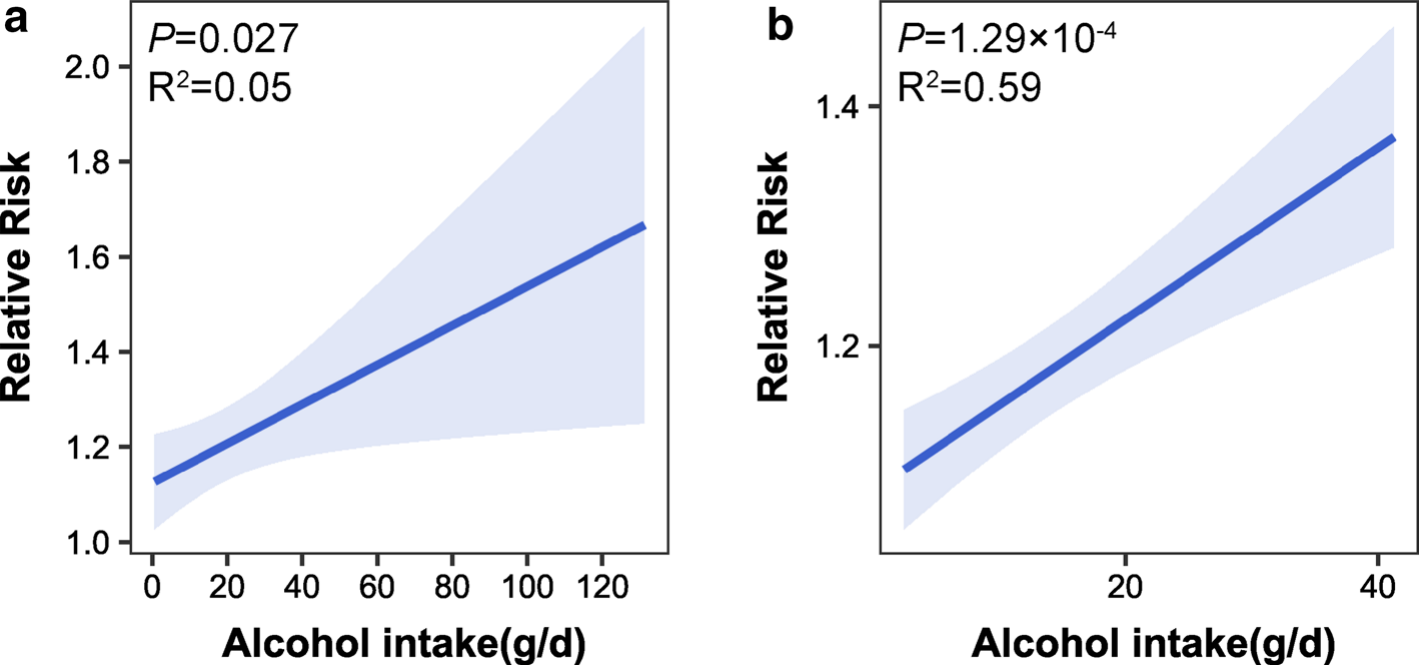
Dose–response relationship between alcohol consumption and breast cancer risk. **a** Incidence risk of overall breast cancer; **b** incidence risk of estrogen receptor positive and/or progesterone receptor positive (ER +/PR +) breast cancer

We included a total of 26 prospective studies with 5,795,688 participants (139,993 cases) for the meta-analysis on breast cancer incidence (Supplementary Fig. 1 and Supplementary Table 1). Risk of bias assessment based on the NOS scale suggested that 23 studies (88.46%) were of high quality and three (11.54%) were of moderate quality (Supplementary Table 2). Figure 2 presents the pooled RR estimates of breast cancer incidence risk for light, moderate and heavy drinkers. Overall, alcohol consumption was significantly associated with increased breast cancer incidence risk with RR estimates of 1.07 (95% CI 1.04, 1.10) for light drinkers, 1.21 (95% CI 1.14, 1.28) for moderate drinkers, and 1.21 (95% CI 1.17, 1.26) for heavy drinkers compared to abstainers. The stratification analyses on menopausal status had consistent dose-dependent trends as the overall analyses, albeit the associations were not significant due to the limited sample size and number of events. When stratified by hormone receptor status, significant associations were observed in both the ER + /PR + and the PR − groups. Restricting to studies conducted in different geographical regions, we found significant associations in studies conducted in Europe, with a RR of 1.08 (95% CI 1.04, 1.12) for light drinkers, 1.17 (95% CI 1.12, 1.23) for moderate drinkers, 1.21 (95% CI 1.17, 1.26) for heavy drinkers in European cohorts, as well as a RR of 1.07 (95% CI 1.00, 1.15) for light drinkers in North American cohorts. When considering alcohol intake as a continuous variable for the dose–response analyses, the overall risk of incident breast cancer increased significantly with about 1.04-fold higher risk per 10 g/d higher intake (*P* = 0.027, R^2^ = 0.05), and the incidence risk of ER + /PR + breast cancer increased about 1.07-fold per 10 g/d increase of alcohol intake (*P* = 1.29 × 10^–4^, R^2^ = 0.59) (Fig. 3). No significant dose–response pattern was observed in other stratification analyses.

### Causal inference—two-sample Mendelian randomisation

#### Genetic predisposition to alcohol consumption and risk of breast cancer incidence

After removing SNPs in LD, 84, 19 and 26 independent IVs were identified to proxy the genetic predisposition to alcohol consumption (drinks per week), AUD and PAU; nine and 11 independent IVs were identified to be associated with AUD and PAU conditioning on drinks per week (Supplementary Table 3). As shown in Table 1, we did not find any suggestive evidence for a causal relationship between genetic predisposition to alcohol drinking and overall breast cancer incidence using the IVW approach. For a one-unit increase in genetically predicted drinks per week (corresponding to 12.5 g ethanol per week), the odds ratio (OR) of overall breast cancer incidence risk was 1.01 (95% CI 0.84, 1.23) using the IVW approach. Results remained unchanged when using the other MR approaches. For AUD and PAU, the ORs were 1.05 (95% CI 0.80, 1.37) and 1.03 (95% CI 0.82, 1.30), respectively. Substantial heterogeneity was reported in the IVW MR analysis and no apparent horizontal pleiotropy was identified using MR Egger regression (*P*_intercept_ = 0.947 for Drinks per Week, *P*_intercept_ = 0.327 for AUD, and *P*_intercept_ = 0.141 for PAU). In the leave-one-out analysis we iteratively removed one SNP at a time and performed IVW using the remaining SNPs, similar null associations were observed. Using MR-PRESSO, we found two outliers among the IVs for drinking per week, three for AUD and two for PAU; the MR estimates remained null after removing outlier SNPs from the analysis (Supplementary Table 4). Based on the meta-analyses results, we calculated the statistical power of MR analysis using the web tool “mRnd” [25]. The MR analysis of drinks per week and overall breast cancer risk had 81% power at significance level of 0.05, indicating that the result was not biased by insufficient power.

**Table 1:** Two-sample MR estimates for the relationship between alcohol consumption and overall breast cancer in the main analyses

Exposure	SNPs	Method	OR (95% CI)	*P*_*Effect*_	*P*_*Heterogeneity*_	*P*_*Intercept*_
Drinks per week	84	IVW	1.01 (0.84, 1.23)	0.883	7.74 × 10^–19^	–
		MR Egger	1.01 (0.72, 1.41)	0.977	4.40 × 10^–19^	0.947
		Weighted median	0.86 (0.71, 1.04)	0.128	–	–
		Simple mode	1.06 (0.59, 1.88)	0.854	–	–
		Weighted mode	0.89 (0.74, 1.07)	0.219	–	–
		MR-PRESSO	0.99 (0.84, 1.16)	0.898	–	–
Alcohol use disorder	19	IVW	1.05 (0.80, 1.37)	0.721	1.06 × 10^–19^	–
		MR Egger	0.89 (0.58, 1.35)	0.586	1.05 × 10^–18^	0.327
		Weighted median	0.90 (0.79, 1.03)	0.138	–	–
		Simple mode	0.94 (0.68, 1.30)	0.718	–	–
		Weighted mode	0.90 (0.79, 1.03)	0.141	–	–
		MR-PRESSO	0.96 (0.77, 1.21)	0.755	–	–
Problematic alcohol use	26	IVW	1.03 (0.82, 1.30)	0.781	2.74 × 10^–17^	–
		MR Egger	0.85 (0.61, 1.19)	0.347	1.67 × 10^–15^	0.141
		Weighted median	0.91 (0.79, 1.04)	0.173	–	–
		Simple mode	1.05 (0.71, 1.54)	0.817	–	–
		Weighted mode	0.90 (0.78, 1.04)	0.159	–	–
		MR-PRESSO	0.97 (0.83, 1.14)	0.721	–	–

When performing MR analyses using IVs for AUD and PAU conditioning on drinks per week, evidence illustrated that genetic predisposition to PAU conditioning on drinks per week led to a higher risk of overall breast cancer incidence, with an OR of 1.76 (95% CI 1.04, 2.99) using IVW and 1.37 (95% CI 1.09, 1.71) using MR-PRESSO (Table 2). For subtypes of breast cancer, we did not observe any causality between general alcohol consumption (drinks per week) and the incidence risk for *HER2*-Enriched-like and triple-negative breast cancer in BCAC, the OR was 1.46 (95% CI 0.92, 2.33) and 0.97 (95% CI 0.72, 1.30), respectively. Combining triple-negative cases in BCAC and affected carriers of *BRCA1* mutation in the Consortium of Investigators of Modifiers of *BRCA1/2* (CIMBA), we found no evidence either, with an OR of 1.01 (95% CI 0.80, 1.26). However, we found causal relationship between AUD and the incidence risk for *HER2*-Enriched-like breast cancer, the OR was 1.60 (95% CI 1.07, 2.39) using the IVW approach and 1.60 (95% CI 1.11, 2.31) using MR-PRESSO (Supplementary Tables 5 and 6). For the sensitivity analyses, we selected four IVs associated with alcohol metabolism and included 64, 15 and 20 IVs for drinks per week, AUD and PAU after removing those associated with potential confounders (Supplementary Tables 7 and 8). Similar null results were observed when restricting to the IVs for alcohol metabolism or independent of potential confounders.

**Table 2:** Two-sample MR estimates for the relationship between alcohol consumption and overall breast cancer in additional analyses

Exposure	SNPs	Method	OR (95% CI)	*P*_*Effect*_	*P*_*Heterogeneity*_
*Using IVs conditionally associated with AUD and PAU but not drinks per week *^*a*^					
Alcohol use disorder	9	IVW	1.70 (0.91, 3.17)	0.096	5.20 × 10^–14^
		MR-PRESSO	1.35 (1.00, 1.83)	0.100	–
Problematic alcohol use	11	IVW	1.76 (1.04, 2.99)	0.036	8.19 × 10^–10^
		MR-PRESSO	1.37 (1.09, 1.71)	0.024	–
*Using IVs associated with alcohol metabolism*^*b*^					
Alcohol metabolism	4	IVW	0.93 (0.57, 1.51)	0.772	5.54 × 10^–4^
		MR-PRESSO	0.93 (0.57, 1.51)	0.791	
*Using IVs not associated with potential confounders*^*c*^					
Drinks per week	64	IVW	1.10 (0.90, 1.34)	0.358	1.10 × 10^–10^
		MR-PRESSO	1.13 (0.90, 1.41)	0.291	–
Alcohol use disorder	15	IVW	0.99 (0.82, 1.19)	0.908	8.85 × 10^–5^
		MR-PRESSO	1.11 (0.84, 1.46)	0.476	–
Problematic alcohol use	20	IVW	1.01 (0.86, 1.19)	0.886	6.68 × 10^–4^
		MR-PRESSO	1.18 (0.93, 1.50)	0.199	–

^a^Selecting IVs for AUD and PAU conditioning on drinks per week (excluding the overlapping or highly correlated genetic IVs (r^2^ > 0.1) with drinks per week);

^b^Selecting IVs associated with alcohol metabolism only;

^c^Excluding IVs associated with obesity related traits (i.e., BMI, waist circumference, hip circumference, and weight), reproductive traits (i.e., age at menarche, age at menopause), smoking, education, and previous breast cancer at the threshold of 5 × 10^–8^ in European ancestry samples

#### Genetically predicted methylation at alcohol related CpG sites and the risk of breast cancer incidence

We identified 363 CpG sites related to alcohol consumption of European ancestry [9]. After removing mQTLs associated with multiple independent CpG sites, 59 CpG sites and 67 corresponding mQTLs were included in the MR analysis (Supplementary Tables 9 and 10). Table 3 and Supplementary Table 11 showed the causal effects of alcohol drinking related blood DNA methylation at each CpG site on overall breast cancer incidence risk. Twelve CpG sites had nominally significant associations with the overall breast cancer incidence risk (*P* < 0.05), and four of them passed the multiple-testing correction (Bonferroni *P* < 0.05/59 = 8.47 × 10^–4^). Three CpG sites (cg03345232, cg26312998, and cg10816169) were located on the CpG islands and the other one (cg03260624) was on the south shelf of the CpG islands (Supplementary Fig. 2). For per SD change in blood DNA methylation at cg03260624 [*CDC7*], cg26312998 [*ZNF318*], cg03345232 [*RIN3*], and cg10816169 [*RP11-867G23.13*], the OR for breast cancer incidence risk was 1.12 (95% CI 1.06, 1.18), 1.04 (95% CI 1.02, 1.06), 1.06 (95% CI 1.03, 1.10), and 1.07 (95% CI 1.03, 1.12), respectively. To investigate whether the mQTLs of these four CpG sites would influence the expression of their mapped gene in breast mammary tissue, we queried the GTEx Portal and found that the mQTL rs13447450 of cg03260624 is an eQTL of the *CDC7* gene (*P* = 2.80 × 10^–5^) and the mQTL rs70953670 of cg26312998 is an eQTL of the *ZNF318* gene (*P* = 0.001) (Table 4 and Supplementary Figs. 3–6). DNA methylation at cg10816169 [*RP11-867G23.13*] was also found to be associated with the subtypes of breast cancer (*P* < 0.05), the incidence risk of *HER2*-Enriched-like breast cancer increased by 22% (OR 1.22, 95% CI 1.03, 1.44) and the incidence risk of triple-negative breast cancer increased by 9% (OR 1.09, 95% CI 1.01, 1.18) in the meta-analysis of BCAC and CIMBA per SD change in blood DNA methylation. (Supplementary Table 12).

**Table 3:** Two-sample MR estimates for per SD of alcohol intake related methylation on overall breast cancer risk (P < 0.05)

CpG site	Chr	Pos	Nearest Gene(s)	Method	SNP	OR (95% CI)	*P* Value
**cg03260624**	**1**	**91,970,722**	***CDC7***	**Wald ratio**	**rs13447450**	**1.12 (1.06, 1.18)**	**4.64 × 10**^**–5**^
**cg26312998**	**6**	**43,337,775**	***ZNF318***	**Wald ratio**	**rs70953670**	**1.04 (1.02, 1.06)**	**6.07 × 10**^**–5**^
**cg03345232**	**14**	**92,981,121**	***RIN3***	**IVW**	**rs12884739, rs77826962**	**1.06 (1.03, 1.10)**	**2.17 × 10**^**–4**^
**cg10816169**	**11**	**66,080,868**	***RP11-867G23.13***	**Wald ratio**	**rs3741368**	**1.07 (1.03, 1.12)**	**5.38 × 10**^**–4**^
cg02282631	5	42,953,543	*–*	Wald ratio	rs36122053	0.94 (0.90, 0.98)	0.006
cg14391586	20	62,681,296	*TCEA2, SOX18*	Wald ratio	rs816943	1.03 (1.01, 1.05)	0.008
cg10045354	11	111,169,427	*COLCA2, COLCA1*	Wald ratio	rs11213823	1.07 (1.01, 1.12)	0.011
cg00883689	2	54,802,904	*SPTBN1*	Wald ratio	rs4455200	1.03 (1.00, 1.05)	0.019
cg05465916	17	7,819,762	*LOC284023*	Wald ratio	rs138596240	1.05 (1.01, 1.09)	0.022
cg14330293	17	1,374,051	*MYO1C*	Wald ratio	rs4411554	1.06 (1.00, 1.12)	0.036
cg08597832	8	144,416,327	*TOP1MT*	Wald ratio	rs2467943	0.97 (0.94, 1.00)	0.047
cg23629150	8	144,416,404	*TOP1MT*	Wald ratio	rs2467933	0.97 (0.95, 1.00)	0.048

Chr, chromosome; Pos, position; OR, odds ratio, breast cancer risk per SD change in DNA methylation at alcohol related CpG sites in blood. The bold ones were those that survived multiple-testing correction (Bonferroni *P* < 8.47 × 10^–4^)

**Table 4:** eQTL effect of significant CpG sites corresponding mQTLs in breast mammary tissue

CpG site	mQTL	Gene	NES	P value	m value
cg03260624	rs13447450	*CDC7*	− 0.186	2.80 × 10^–5^	0.972
cg03345232	rs12884739	*RIN3*	0.060	0.100	0.455
cg03345232	rs77826962	*RIN3*	NA	NA	NA
cg10816169	rs3741368	*RP11-867G23.13*	− 0.077	0.100	0.037
cg26312998	rs70953670	*ZNF318*	0.167	1.00 × 10^–3^	1.000

NES, normalized effect size. m value, the posterior probability that an eQTL effect exists in each tissue tested in the cross-tissue meta-analysis. Small m value (e.g., < 0.1), the tissue is predicted to NOT have an eQTL effect; large m value (e.g., > 0.9), the tissue is predicted to Have an eQTL effect; otherwise, the prediction of the existence of an eQTL effect is ambiguous

## Discussion

We performed an updated meta-analysis assessing the observational association between alcohol intake and the risk of breast cancer incidence. We then made causal inferences based on the genetic predisposition to alcohol consumption and pathological drinking behaviours proxied by SNPs from two GWASs [7, 8]. Furthermore, we examined the causal effects of genetically predicted methylation at alcohol related CpG sites on the risk of breast cancer incidence.

Meta-analysis of 26 prospective studies confirmed a positive association between alcohol intake and breast cancer incidence, which was dose-dependent. In the stratification analyses, there were significant associations between alcohol drinking and the incidence risk of breast cancer subtypes regardless of the hormone receptor status. In contrast to Sun et al. [12], we did not identify a significant association between alcohol drinking and breast cancer incidence risk in postmenopausal women. Sun et al. included eight studies on postmenopausal breast cancer, while there were only three studies on postmenopausal breast cancer included in our study, making our study less-powered to discover the association. Dose–response analysis supported the existence of a significant linear association. The coefficients of determination (*R-* squared) in these dose–response analyses were not high, which might be because most of the included studies reported risk estimates of alcohol consumption at the light and moderate drinking levels and only few studies reported risk estimates at heavy drinking levels, or there might be other uncontrolled confounders and biases.

To make causal inference, we conducted two-sample MR analyses using genetic variants derived from published GWAS of alcohol consumption and pathological drinking behaviours [7, 8]. The phenotype of “drinks per week” represents the general exposure of alcohol consumption, and other two phenotypes “AUD” and “PAU” reflects pathological drinking behaviours. We did not find causal association for any of the three alcohol-related phenotypes in the main analyses. Our MR findings were consistent with the other three published MR analyses in which they did not observe any causal effect either. Zhu et al. and Larsson et al. used the same genetic instruments from the GWAS of “drinks per week” including both sexes [6, 26]. In contrast, Ong et al. performed two separate GWASs using the estimated alcohol quantity in both sexes and females only to identify genetic instruments for MR analysis, and the null results were not modified by using female-specific effect estimates for alcohol drinking [27]. However, causal effect was observed between PAU and breast cancer incidence risk when conditioning on alcohol consumption, indicating that the carcinogenic effect of alcohol might act accumulatively through a severe pathological drinking behaviour.

Recent EWAS showed that alcohol consumption can affect DNA methylation in both blood and tissues, indicating that DNA methylation at certain sites could act as a marker for the exposure to alcohol [10]. Based on findings from an EWAS conducted by Liu et al. [9], we identified CpG sites in blood related to alcohol consumption and selected their corresponding independent mQTLs as genetic IVs to perform MR analysis. Epigenetic MR analysis found four significant CpG sites after Bonferroni correction, including cg03260624 near *CDC7* gene, cg10816169 near *ZNF318* gene, cg03345232 near *RIN3* gene, and cg26312998 near *RP11-867G23.13* gene, where genetically predicted methylation was causally associated with increased breast cancer risk. Furthermore, the mQTL rs13447450 of CpG site cg03260624 and rs70953670 of CpG site cg26312998 are strong eQTLs of the *CDC7* gene and the *ZNF318* gene in breast mammary tissue, respectively, indicating that the effect of changed methylation at cg03260624 and cg26312998 on breast cancer might be mediated by gene expression.

*CDC7* is a protein coding gene, which is essential for the initiation of DNA replication during cell division. It has been reported that overexpression of *CDC7* is associated with tumorigenesis [28]. The increased expression of *CDC7* has been linked to *HER2*-Enriched and triple-negative subtypes, accelerated cell cycle progression, arrested tumour differentiation and genomic instability during the tumorigenesis of mammary tissue and led to poorer disease-free survival [29]. By targeting *CDC7*, *p53*-mutant *HER2*-overexpressing and triple-negative breast cancer cells undergo an abortive S phase and apoptotic cell death, suggesting the potential therapeutic effect of *CDC7* in *p53*-mutant breast cancers [29]. Moreover, in a research conducted by Cheng et al., it was found that most cases of oral squamous cell carcinoma patients had overexpression of *CDC7*, and poorer outcome was observed among patients with higher expression of *CDC7* [30]. It has been speculated that *CDC7* would inhibit genotoxin-induced apoptosis and protect cancer cells upon DNA damage response, so that it would enhance chemotherapy resistance [30]. Meanwhile, Melling et al. discovered that *CDC7* was also highly expressed among colorectal cancer patients and interacted with the expression of *p53* [31].

Though previous studies had reported the impact of *CDC7* on multiple cancers, the other two genes and the lncRNA are novel in relation to genetic predisposition to cancer, with the potential to be new targets in the treatment and prevention of breast cancer. *ZNF318* is a member of the zinc finger protein family, encoding the Cys2His2-type [32]. Acting as transcription factors, several C2H2-types have been found to be involved in cancer growth, apoptosis, invasion and metastasis. For example, *ZBP89*, also known as *ZNF148*, has been reported to have oncogenic functions in breast cancer, melanoma and gastric cancer, but repressing cell proliferation and inducing apoptosis in colorectal cancer. Generally, the zinc finger family participated in all the principal pathways of cancer progression and could act as oncogenes or tumour suppressor genes in different contexts [33]. No previous study has reported the role of *ZNF318* in breast cancer development. *RIN3* is a protein coding gene of the *RAS* and *RAB* interactor family, which was discovered functioning as a guanine nucleotide exchange for RAB5B and RAB31, involved in the vesicle transportation [34]. A genome-wide association study found that mutation at *RIN3* is a novel risk locus for Alzheimer’s Disease due to the dysfunction of endocytic trafficking and had effects on the development of Paget’s Disease of Bone [35, 36]. But the relationship of *RIN3* and breast cancer has not been previously reported. Though mapped to lncRNA *RP11-867G23.13*, CpG site cg10816169 was also close to the gene *CD248*, which encoded transmembrane glycoprotein functioning as a receptor in tumour angiogenesis. By cross-talking with both pro- and anti-angiogenic signals and extracellular matrix components, and participating in dynamic vascular remodelling, *CD248* could facilitate tumour growth [37]. Besides, humanized monoclonal antibody ontuxizumab had been developed targeting *CD248* and is investigated in clinical trials for colorectal cancer, melanoma and sarcoma [38–40].

Our study has several strengths and limitations. We examined the causal relationship between alcohol and breast cancer incidence comprehensively by aggregating both quantitative and qualitative traits of alcohol consumption, with drinks per week reflecting the general effects of drinking, AUD and PAU representing pathological drinking behaviours. We also appraised the causal effects of genetically predicted methylation at alcohol associated CpG sites on the incidence risk of breast cancer under the framework of MR, which can strengthen causal inference by minimizing unobserved confounding and diminishing reverse causality [41]. However, our study is also limited. Without access to the full summary level data of methylation across the whole epigenome, we could not perform MR to explore the causal effect of alcohol consumption on DNA methylation but derived CpG sites associated with alcohol drinking from a cross-sectional EWAS. Our epigenetic MR analyses were based on the methylation level of whole blood DNA instead of breast tissue DNA [9, 13]. Future epigenetic studies with breast tissue-specific DNA methylation data is worth doing to validate the observed associations between these alcohol related CpG sites and breast cancer incidence risk. Additionally, it should be noted that GWAS of drinks per week was conducted among participants restricted to active drinkers, while the GWAS of AUD and PAU was performed among severe drinkers who were diagnosed of AUD and PAU and controls which were non-drinkers. The inclusion of only active drinkers in the GWAS of drinks per week might influence the MR result towards the null, as the effect of one drink per week might not be as strong as that of severe drinkers who were diagnosed of AUD and PAU versus non-drinkers. When performing MR analysis of AUD and PAU conditioning on drinks per week, we did find the causal effect of PAU on breast cancer risk. The EWAS measured the methylation changes related to alcohol consumption in populations of drinkers versus non-drinkers, and the epigenetic MR based on EWAS found significant effects on breast cancer risk. Taken all these points together, the inconsistency between the null finding from the MR of drinks per week and the significant findings from the MR of PAU and the epigenetic MR might be partially attributed to the different study populations or the differences how alcohol consumption was measured.

In conclusion, with an updated meta-analysis of prospective studies, our study re-affirmed the dose–response association between alcohol intake and breast cancer incidence. Evaluating the causal effect using the two-sample MR approach, the pathogenic effect of alcohol on breast cancer could be due to pathological drinking behaviour and epigenetic modification at several CpG sites, which could be potential intervention targets for breast cancer prevention.

## Supplementary Information

Below is the link to the electronic supplementary material.

Supplementary file 1 (DOCX 2197 KB)

Supplementary file 2 (XLSX 117 KB)

## Data Availability

All data analysed during this study are included in this published article and its supplementary information files, except for GWAS summary-level data of breast cancer which is available on http://bcac.ccge.medschl.cam.ac.uk/bcacdata/oncoarray/oncoarray-and-combined-summary-result/.
