# Supplementary material for: Alcohol consumption, blood DNA methylation and breast cancer: a Mendelian randomisation study
Source: Eur J Epidemiol. 2022 Jun 16;37(7):701–12. doi: 10.1007/s10654-022-00886-1 (PMC9329409; doi:10.1007/s10654-022-00886-1)
Supplement: Supplementary file 1 — (DOCX 2197 KB) [file 10654_2022_886_MOESM1_ESM.docx]

**Supplementary Methods**

**Meta-analysis of prospective studies**

**Literature search**

We carried out a comprehensive literature search in MEDLINE and EMBASE databases (both from the OVID interface) from inception to March 22, 2021, using keywords of alcohol, breast cancer, and cohort. The detailed search strategy can be seen below. Reference lists of all included articles were checked to identify any additional eligible studies.

MEDLINE: [alcohols OR ethanol OR drinking OR drinking behavior OR (exp alcohol drinking) OR (exp alcoholic beverages) OR (exp alcohol-related disorders) OR ((alcohol and (consumption or drinking or intake)) or (alcohol abuse) or (alcoholic beverage*)).mp.] AND [(exp breast neoplasms) OR ((breast or mammar*) and (cancer or neoplas* or malign* or tumor* or tumour* or carcino* or adenocarcinoma or sarcoma or lymphoma or leiomyosarcoma or dics or duct* or lobul*)).mp.] AND [(exp cohort studies) OR (cohort$.tw.) OR (controlled clinical trial.pt.)]

EMBASE: [alcohol OR alcohol consumption OR drinking OR drinking behavior OR alcoholism OR (exp alcohol abuse) OR (exp alcoholic beverages) OR ((alcohol and (consumption or drinking or intake)) or (alcohol abuse) or (alcoholic beverage*)).mp.] AND [(exp breast cancer) OR ((breast or mammar*) and (cancer or neoplas* or malign* or tumor* or tumour* or carcino* or adenocarcinoma or sarcoma or lymphoma or leiomyosarcoma or dics or duct* or lobul*)).mp.] AND [cohort analysis OR longitudinal study OR prospective study OR follow up OR (cohort$.tw.)]

Studies meeting the following criteria were eligible for inclusion: (i) examining the relationship between alcohol consumption and breast cancer incidence in prospective design (i.e., cohort studies, nested case-control studies and case-cohort studies); (ii) reporting findings expressed as relative risk (RR) or hazard ratio (HR), or reporting sufficient data to compute them for at least two levels of alcohol consumption vs non-drinkers (with or without occasional drinkers); (iii) reporting standard errors (se) or confidence intervals (CIs) of effect estimates or providing sufficient data to calculate them. We excluded studies examining the risk of breast cancer mortality; involving patients with prior breast cancer history; evaluating the synergic or interaction effects between alcohol and other factors (genetic variants, drugs, *etc*.); or focusing on a specific type of alcoholic beverage only.

**Data abstraction**

The retrieved articles were independently reviewed by two authors to determine the eligibility for inclusion in the meta-analysis. Doubts and disagreements were resolved by consensus among all the investigators. When the results of the same cohort were published in more than one paper, only the most recent one or the one with the most comprehensive data was included. For each independent study, information of the first author, year, study population, follow-up duration, RR or HR estimates with corresponding 95% CIs of different exposure levels, adjusted confounding factors, the number of cases, and subjects at risk or person-years were extracted. We also recorded whether ex-drinkers and occasional drinkers were included in the reference category. The risk of bias of the included studies was assessed using the Newcastle–Ottawa scale (NOS) [1].

Since different studies used different units of measurement, we set grams per day (g/day) as a standard, considering one drink as 12.5g, one ml as 0.8g, and one ounce as 28g of ethanol[2], unless the original studies had specified it. If the levels of consumption were reported by a range, the midpoints were assigned; and for an open-ended upper category, three-quarters of the length of the last level plus the lower bound was calculated[2, 3]. We divided alcohol consumption into three categories, considering ≤12.5g, 12.5-50g and > 50g alcohol per day as light, moderate and heavy drinking, respectively. If the original studies reported two or more transformed risk estimates for a single dose category, we used the method developed by Hamling *et al* to combine them into one single estimate with its 95% CIs [4].

**Two-sample Mendelian randomisation**

Mendelian randomisation (MR) design provides a cost-effective analogy to a randomized controlled trial by using genetic variants as proxies to test the causality of an association between exposure and outcome of interest. Here we applied inverse-variance weighted MR approach as the main analysis, and the simple mode, Egger, weighted median and weighted mode as sensitivity analyses to explore the robustness of the findings. Details of these MR approaches, including their different assumptions, are explained as below.

Mean-based methods: The inverse variance weighted (IVW) MR and Egger MR provide two mean-based estimators. The IVW MR approach assumes that variants exhibit no horizontal pleiotropy, while Egger regression relaxes the horizontal pleiotropy assumption further by allowing a non-zero intercept which essentially allows overall horizontal pleiotropy to be directional, where its total effect influences the outcome in a specific direction. Egger regression further allows heterogeneity around the slope having accounted for overall directional horizontal pleiotropy, as long as the horizontal pleiotropy effects are not correlated with the SNP-exposure effects (also known as the INSIDE assumption).

Median-based methods: This analytical approach takes the median effect of all available instruments. The sample median method requires that half the instruments need to be valid to obtain unbiased estimate. The weighted median method allows stronger instruments to contribute more towards the estimate and obtain an estimate by weighting the contribution of each instrument by the inverse of its variance.

Mode-based methods: The mode-based estimator clusters the instruments into groups based on similarity of causal effects and returns the final causal effect estimate based on the cluster that has the largest number of instruments. This provide two mode-based estimators: the simple mode is the unweighted mode of the empirical density function of causal estimates, the weighted mode is weighted by the inverse variance of the outcome effect.

MR-PRESSO: The MR-PRESSO is applied for the global test, outlier test, and distortion test using the MR pleiotropy residual sum and outlier (MR-PRESSO) R package. Specifically, the global test detects horizontal pleiotropy among the MR instruments; the outlier test corrects for horizontal pleiotropy via outlier removal; the distortion test identifies significant distortion in the causal estimates before and after outlier removal.

**References**

1. Wells G, Shea B, O'Connell J. The Newcastle-Ottawa Scale (NOS) for Assessing The Quality of Nonrandomised Studies in Meta-analyses. Ottawa Health Research Institute Web site. 2014;7.

2. Bagnardi V, Rota M, Botteri E, et al. Alcohol consumption and site-specific cancer risk: a comprehensive dose-response meta-analysis. British journal of cancer. 2015;112(3):580–93. doi:10.1038/bjc.2014.579

3. Patra J, Bakker R, Irving H, Jaddoe VWV, Malini S, Rehm J. Dose-response relationship between alcohol consumption before and during pregnancy and the risks of low birthweight, preterm birth and small for gestational age (SGA)-a systematic review and meta-analyses. BJOG : an international journal of obstetrics and gynaecology. 2011;118(12):1411–21. doi:10.1111/j.1471-0528.2011.03050.x

4. Hamling J, Lee P, Weitkunat R, Ambühl M. Facilitating meta-analyses by deriving relative effect and precision estimates for alternative comparisons from a set of estimates presented by exposure level or disease category. Statistics in medicine. 2008;27(7):954–70. doi:10.1002/sim.3013


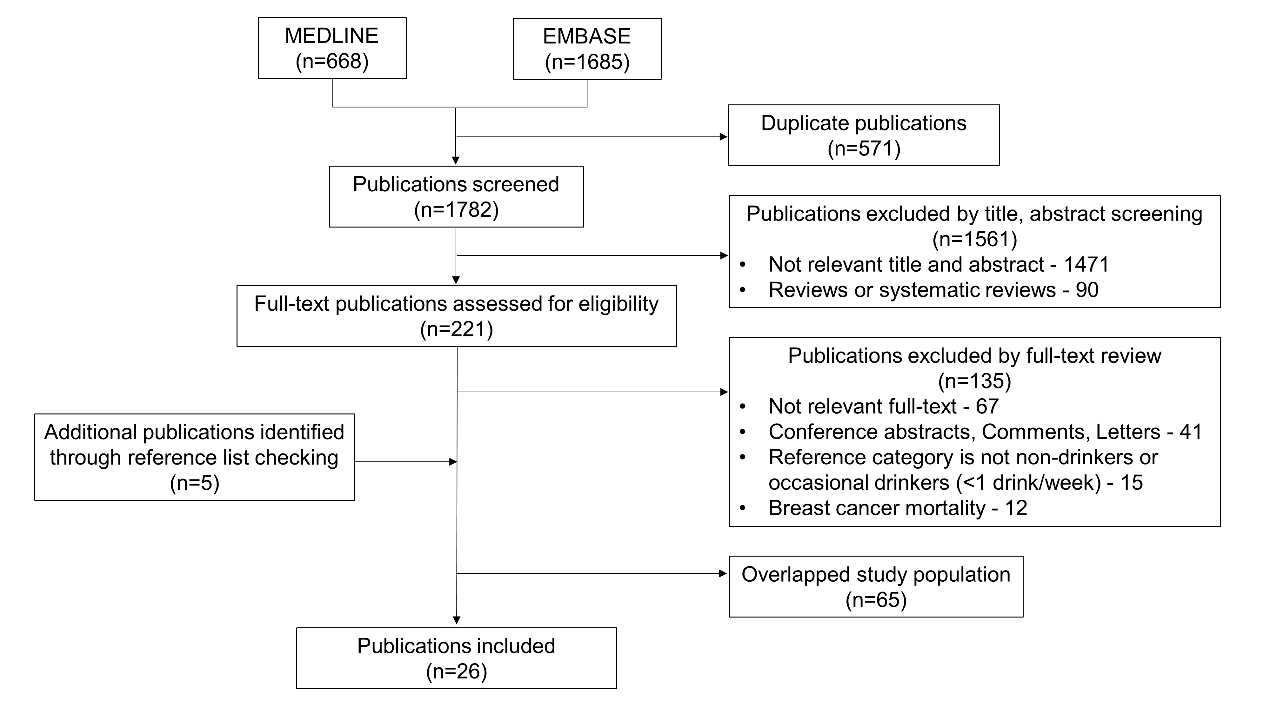


**Supplementary Figure 1.** Flowchart of selection of studies included in the meta-analysis.

**
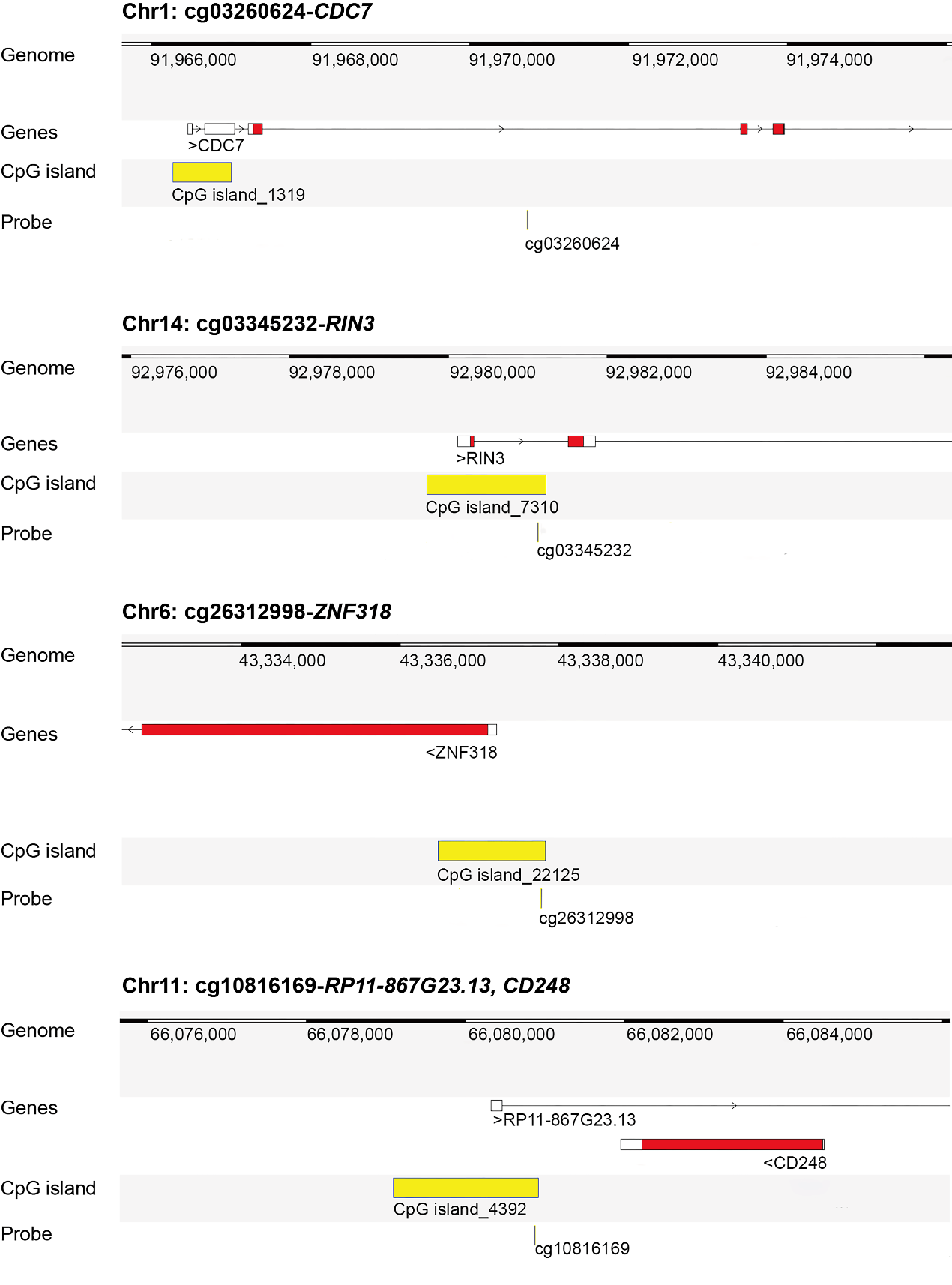
**

**Supplementary Figure 2.** Regional plots of CpG sites and mapped genes.


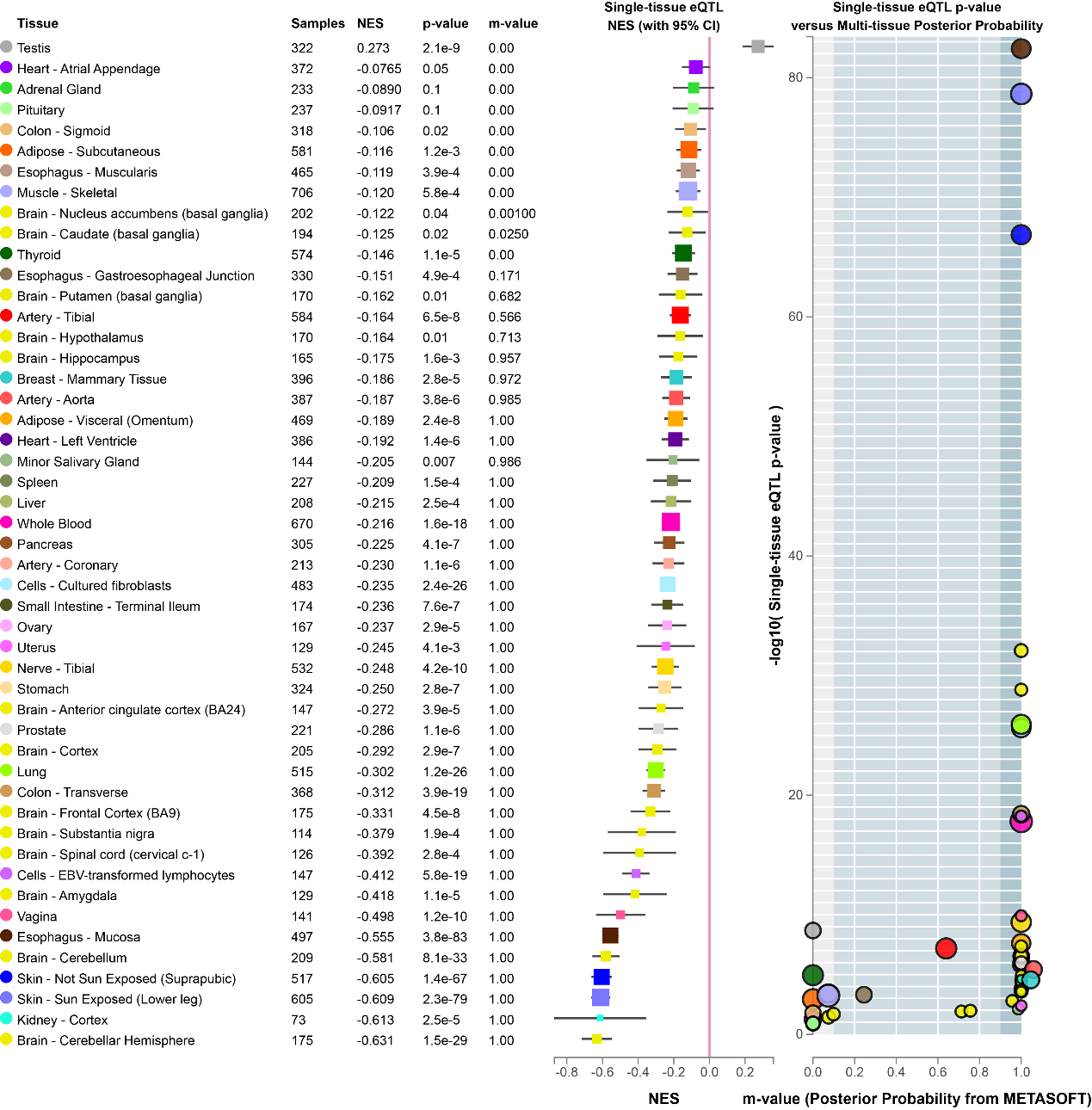


**Supplementary Figure 3.** Single-tissue eQTL plot of rs13447450 (cg03260624, *CDC7*). NES, normalized effect size. m value, the posterior probability that an eQTL effect exists in each tissue tested in the cross-tissue meta-analysis. Small m value (e.g., <0.1), the tissue is predicted to NOT have an eQTL effect; large m value (e.g., >0.9), the tissue is predicted to Have an eQTL effect; otherwise, the prediction of the existence of an eQTL effect is ambiguous.


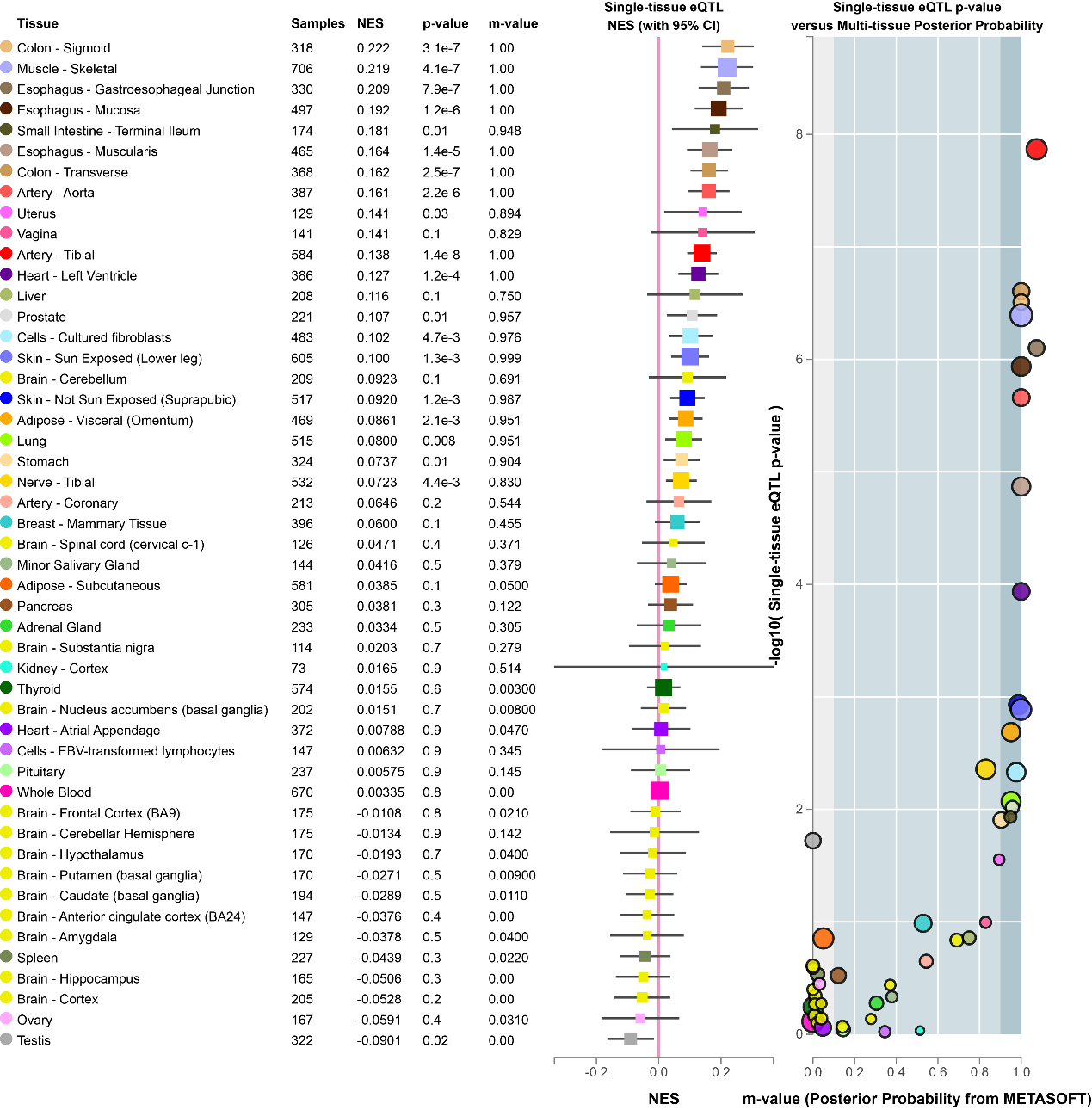


**Supplementary Figure 4.** Single-tissue eQTL plot of rs12884739 (cg03345232, *RIN3*). NES, normalized effect size. m value, the posterior probability that an eQTL effect exists in each tissue tested in the cross-tissue meta-analysis. Small m value (e.g., <0.1), the tissue is predicted to NOT have an eQTL effect; large m value (e.g., >0.9), the tissue is predicted to Have an eQTL effect; otherwise, the prediction of the existence of an eQTL effect is ambiguous.

**
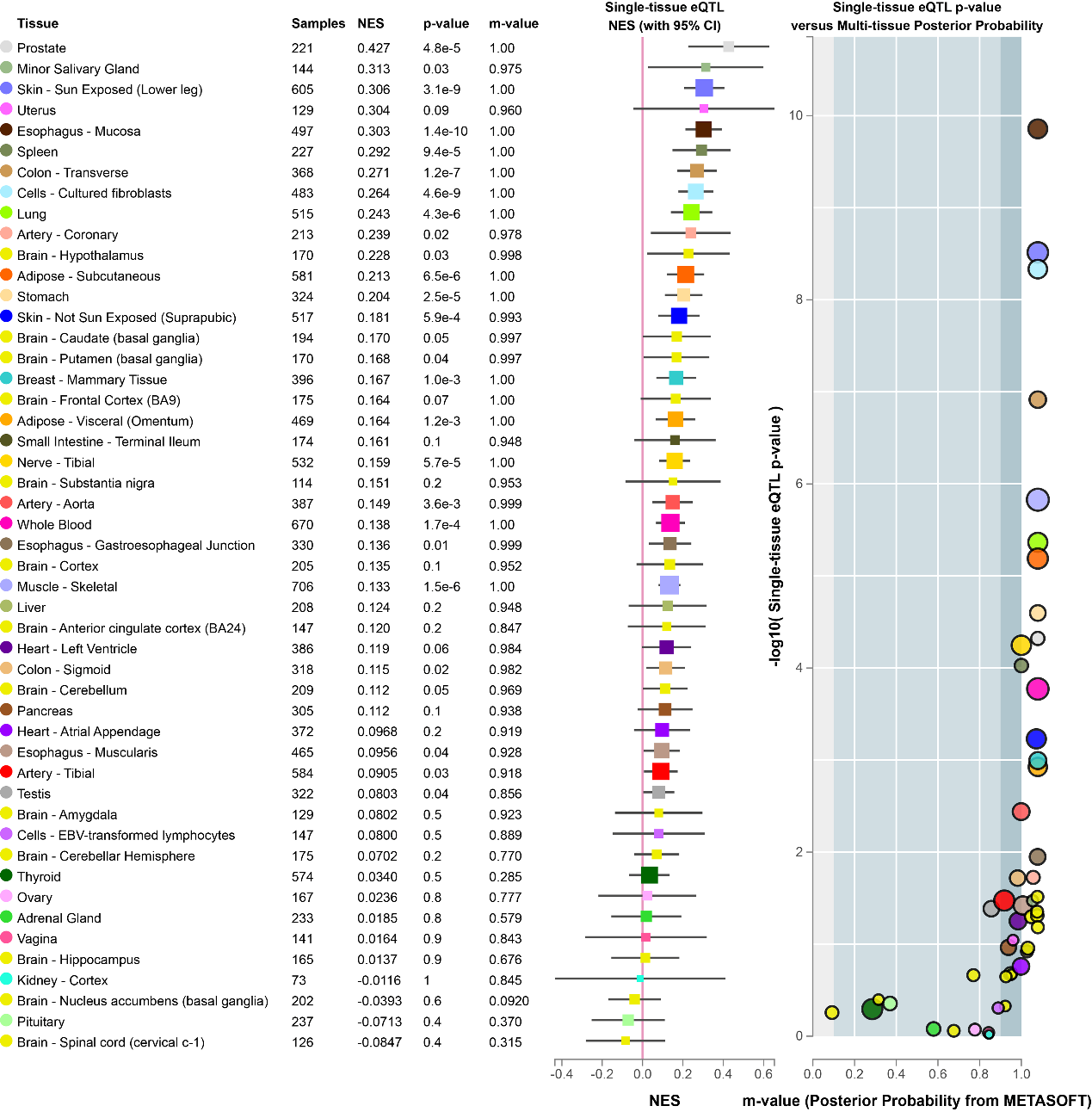
**

**Supplementary Figure 5.** Single-tissue eQTL plot of rs70953670 (cg26312998, *ZNF318*). NES, normalized effect size. m value, the posterior probability that an eQTL effect exists in each tissue tested in the cross-tissue meta-analysis. Small m value (e.g., <0.1), the tissue is predicted to NOT have an eQTL effect; large m value (e.g., >0.9), the tissue is predicted to Have an eQTL effect; otherwise, the prediction of the existence of an eQTL effect is ambiguous.

**
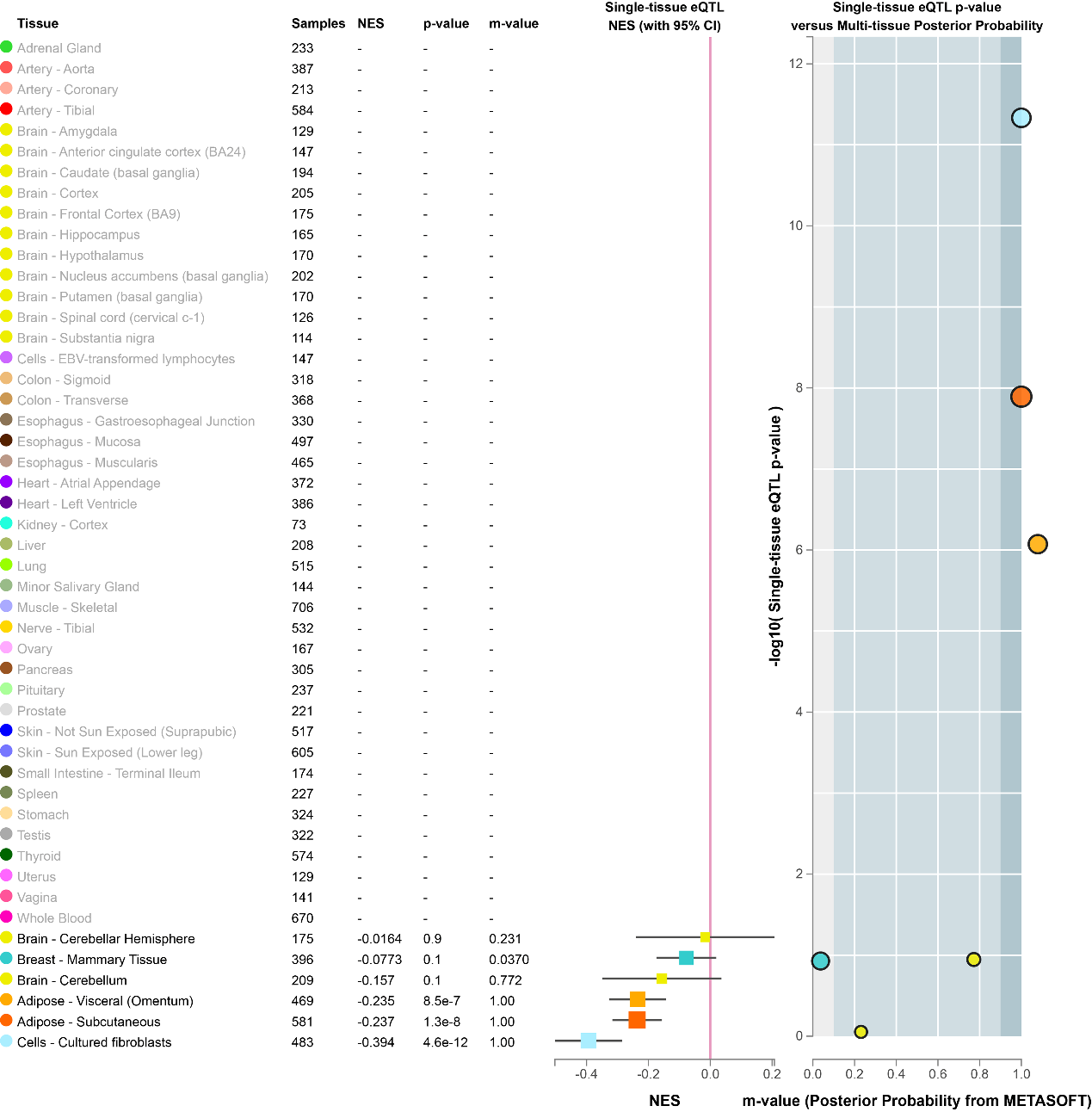
**

**Supplementary Figure 6.** Single-tissue eQTL plot of rs3741368 (cg10816169, *RP11-867G23.13*). NES, normalized effect size. m value, the posterior probability that an eQTL effect exists in each tissue tested in the cross-tissue meta-analysis. Small m value (e.g., <0.1), the tissue is predicted to NOT have an eQTL effect; large m value (e.g., >0.9), the tissue is predicted to Have an eQTL effect; otherwise, the prediction of the existence of an eQTL effect is ambiguous.
